# Immunotherapy for autoimmune encephalitis

**DOI:** 10.1038/s41420-025-02459-z

**Published:** 2025-04-29

**Authors:** Lufeng Cheng, Bingyang Jia, Chuanlei Wang, Qingxi Fu, Lingyan Zhou

**Affiliations:** 1 Department of Neurology, Linyi People’s Hospital, Linyi, Shandong China; 2 Department of Cardiothoracic Surgery, Linyi People’s Hospital, Linyi, Shandong China; 3 Department of Neurology, Xuzhou Medical University, Xuzhou, China; 4 Department of Neurology, Shandong Provincial Hospital Affiliated to Shandong First Medical University, Jinan, Shandong China

**Keywords:** Immunosuppression, Diseases of the nervous system

## Abstract

Autoimmune encephalitis (AE) is increasingly recognized as a cause of brain disorders that greatly benefit from immunotherapy. Starting treatment quickly and increasing the use of immunotherapy can lead to better results for AE patients. Currently, there are standardized treatment guidelines for treating AE. First-line therapy includes intravenous corticosteroids, plasma exchange, and intravenous immunoglobulin. Second-line therapy involves rituximab, cyclophosphamide, mycophenolate mofetil, and azathioprine. Third-line therapy uses agents that deplete plasma cells (bortezomib, daratumumab, and obinutuzumab), drugs that modulate cytokines (tocilizumab, anakinra, tofacitinib, and interleukin-2), and treatments that target intrathecal immune cells (intrathecal methotrexate). This review aims to summarize the immunotherapeutic strategies available for treating AE and provide an update on refractory AE.

## Facts

- Autoimmune encephalitis (AE) is mediated by autoantibodies targeting neuronal surface or synaptic proteins.
- Immunotherapy is the cornerstone of AE treatment, classified into first-line, second-line, and third-line therapies.
- A subset of AE patients remains refractory to standard immunotherapy, necessitating novel treatment approaches.
- Long-lived plasma cells and intrathecal immune responses contribute to disease persistence, posing challenges to current therapeutic strategies.
- Large-scale randomized controlled trials (RCTs) are essential to refine treatment protocols and improve long-term outcomes.

## Open questions

- What biomarkers can predict treatment response and long-term prognosis in AE?
- How can immunotherapy be personalized to enhance efficacy while minimizing adverse effects?
- What is the optimal treatment sequence and combination for refractory AE?
- How does the timing of immunotherapy (early vs. delayed intervention) influence long-term neurological outcomes?

## Introduction

Autoimmune encephalitis (AE) represents a group of diseases characterized by autoantibodies against the neuronal cell surface or synaptic proteins in the serum and cerebrospinal fluid (CSF). Different antibody types, IgG subclasses and epitope specificities lead to different pathogenic effects [1]. Among the most commonly found proteins are antibodies against NMDA, GABA(B), and glycine receptors, as well as proteins such as CASPR2, DPPX, and LGI1 [2]. The clinical manifestations of AE are diverse and include abnormal psychiatric behaviour, cognitive dysfunction, speech dysfunction, seizures, movement disorders, decreased levels of consciousness, autonomic dysfunction, and central hypoventilation [3].

**Fig. 1:**
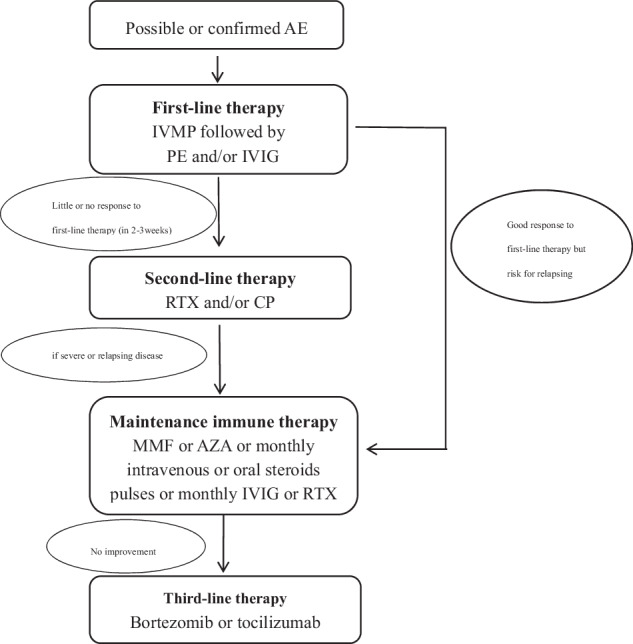
Immunotherapy flowchart. AE autoimmune encephalitis, IVMP intravenous methylprendisolone, IVIG intravenous immunoglobulin, PE plasma exchange, RTX rituximab, CP cyclophosphamide, MMF mycophenolate mofetil, AZA azathioprine.

A recent epidemiological study from the United States revealed that AE is as common as an infectious cause of encephalitis, with a prevalence of 13.7 per 100,000 [4]. Early recognition and immunotherapy of AE are associated with better clinical outcomes [5]. The consensus supports a framework of first-line, second-line, and maintenance immunotherapies [6]. This review aims to provide a summary and discussion of the immunotherapeutic strategies available for treating AE (Fig. 1, Table 1). We elaborate in detail on the first-line and second-line immunotherapies that are currently widely used and focus on potential treatment methods for refractory AE.

**Table 1:** Overview of immunotherapy in autoimmune encephalitis.

Therapy	Name	Mechanism	Regimen
First-line therapy	Corticosteroids	Nonspecific cytokine inhibitor	Methylprednisolone 1000 mg/day, intravenous infusion for 3 days; then reduced to 500 mg/day, intravenous infusion for another 3 days. Thereafter, the dosage can be tapered to 40–80 mg/day, intravenous infusion for 2 weeks; or switched to oral prednisone at a dose of 1 mg/kg/day for 2 weeks; followed by a reduction of 5 mg every 2 weeks.
	Intravenous immunoglobulin	Polyclonal IgG, with immunomodulatory and anti-inflammatory effects	Each treatment course: total dose of 2 g/kg, administered intravenously over 3 to 5 days. Intensive first-line immunotherapy: can be reapplied every 2 to 4 weeks.
	Plasma exchange	Clearing pathogenic antibodies from the blood	Each treatment course: 5–7 plasma volume exchanges, each equivalent to 1–2 plasma volumes, to be carried out over 7–10 days.
Second-line therapy	Rituximab	Anti-CD20 monoclonal antibody, depleting B cells	Conventional regimen: 375 mg/m² (up to a maximum of 1 g), once a week for 4 consecutive weeks. Dose reduction regimen: a total of 600 mg (100 mg on day 1, and 500 mg on day 2) or a total of 400 mg (100 mg per dose, once a week for 4 consecutive weeks).
	Cyclophosphamide	Alkylating agent, cross-linking DNA strands and inhibiting protein synthesis	750 mg/m^2^ (up to a maximum of 1,500 mg), once every 4 weeks, for 6 or more doses, or until disease remission.
	Mycophenolate mofetil	Inhibitors of inosine monophosphate dehydrogenase, suppressing the activity of B and T lymphocytes as well as plasma cells	1000 to 2000 mg/day, divided into 2 to 3 times oral administration of 3000 mg/day; the induction phase dosage can go up to 2,500 to 3,000 mg/day.
	Azathioprine	6-Mercaptopurine analogues, inhibiting nucleic acid synthesis and modulating the immune system	100 mg/day, generally taken orally in two divided doses.
Third-line therapy	Bortezomib	26S proteasome inhibitor, depleting plasma cells	Subcutaneous injection of 1.3 mg/m^2^, twice weekly for 2 weeks (days 1, 4, 8, and 11), followed by a 10-day rest, generally, 1–6 courses of treatment are used.
	Daratumumab	Monoclonal antibody against CD38, inducing plasma cell apoptosis	Intravenous infusion, 16 mg/kg per cycle.
	Obinutuzumab	Type II anti-CD20 monoclonal, depleting B cells antibody	Intravenous infusion, 1000 mg.
	Tocilizumab	Monoclonal antibody against IL-6 receptor, inhibiting the signal transduction pathways triggered by IL-6	Conventional regimen: 8 mg/kg per dose (up to a maximum of 800 mg per dose), once every 4 weeks, for 6 or more doses. Dose reduction regimen: 2 to 6 mg/kg per dose, once every 4 weeks.
	Anakinra	Interleukin-1 antagonist, reducing inflammation	Subcutaneous, 100 mg daily.
	Tofacitinab	JAK enzymes inhibitor, blocking inflammatory signalling pathways	Oral, 5 mg twice daily.
	Low-dose IL-2	Stimulating T-regulatory lymphocytes	Subcutaneous injection of 1.5 million IU/day for 5 days, followed by three 5-daycourses of 3 million IU/day at weeks 3, 6, and 9.
	Intrathecal methotrexate	Folate synthesis inhibitor	Intrathecal, 8-12 mg, once a week for 3 to 4 weeks consecutively.

## First-line therapies

### Corticosteroids

Intravenous corticosteroids are a first-line treatment for various autoimmune diseases of the nervous system. In AE, corticosteroids can improve cognition and reduce seizure frequency, such as in faciobrachial dystonic seizures (FBDSs) [7]. The dosing and weaning regimen of corticosteroids depends on the type of autoantibody involved. For example, in anti-NMDAR encephalitis, corticosteroid induction without tapering can be successful, whereas in LGI1 encephalitis, a prolonged course of corticosteroids is necessary [8]. Corticosteroids possess potent anti-inflammatory and immunosuppressive properties. Methylprednisolone (MP) induces the expression of the anti-inflammatory cytokines IL-10 and TGF-β and suppresses the expression of proinflammatory cytokine genes such as IFN-γ and TNF-α. MP is effective in the treatment of central nervous system disorders by suppressing the inflammatory immune response and preventing immune cells from migrating from the periphery into the CNS [9].

In a study on LGI1 encephalitis by Rodriguez A et al., corticosteroids were used in at least 90% of patients, with most showing improvement, and the results also suggested that acute treatment with corticosteroids may be more effective than IVIg in improving acute outcomes [10]. Another study by Dong Li et al. revealed that a decreased level of consciousness at disease onset may be associated with a prolonged corticosteroid course. They also noted that the benefits of oral corticosteroid treatment may not persist beyond one year and could increase the risk of adverse effects. The long-term use of corticosteroids can lead to side effects such as weight gain, hypertension, hyperglycaemia, and opportunistic infections. To maximize the effectiveness and safety of treatment, a course of 3–12 months of corticosteroids is recommended [11].

### Intravenous immunoglobulin (IVIG)

Derived from pooled polyclonal IgG from donor serum, IVIG has been proven to be an effective anti-inflammatory and immunomodulatory treatment for several neurological diseases [12]. In treating AE, IVIG is often recommended in conjunction with corticosteroids [13]. In a study by Tao-Ran Li et al. on anti-GAD65 encephalitis, IVIG appeared to have a slightly better treatment effect than intravenous MP (IVMP) among patients with stiff-person syndrome or cerebellar ataxia [14]. IVIG mitigates inflammatory responses by acting on cells and processes involved in both innate and adaptive immune responses. This includes inhibiting the activation of monocytes and macrophages; inducing anti-inflammatory cytokines such as the IL-1 receptor antagonist, TGF-β, and IL-10; exerting cytotoxic effects on neutrophils; and improving the clearance of pathogenic antibodies by saturating the FcRn receptor [15]. Common adverse effects of IVIG include mild to moderate headache, fever, chills, chest or back pain during the first few hours of infusion, and postinfusion fatigue. In rare cases, thromboembolic events, such as myocardial infarction, stroke, and pulmonary embolism, occur after IVIG treatment.

### Plasma exchange (PE)

PE involves removing blood plasma from the body, separating it, and replacing it with an appropriate fluid, commonly a human albumin solution. It is a relatively safe, tolerable, and effective treatment for autoimmune neurological diseases. Typically, PE is utilized after steroids and IVIG. Patients receiving first-line immune therapy combining PE with steroids (with or without IVIG) have higher recovery rates than those receiving other treatments without steroids [16]. The American Society for Apheresis (ASFA) concluded that PE is probably effective in treating AE, such as LGI1 and CASPR2 encephalitis [17]. A pilot study indicated that PE was more effective for patients with neuronal surface autoantibodies (NMDA, LGI1, CASPR, mGluR5) than for those with intracellular-synaptic antigens (Hu, GAD) [18]. The complications of PE include symptomatic hypocalcemia, allergic reactions, fluid-electrolyte disturbances, hypotension, infection, and catheter placement issues [19]. Notably, the use of PE shortly after IVIG or rituximab should be performed carefully because these therapeutic antibodies can be removed after PE.

## Second-line therapies

### Rituximab (RTX)

RTX is a first-generation chimeric monoclonal antibody that targets the B-cell-specific antigen CD20, depletes B cells and impacts the entire spectrum of B cell functions. It was initially approved by the Federal Drug Administration (FDA) in 1997 for treating non-Hodgkin’s lymphoma [20]. RTX can eliminate 90% of circulating B cells in the blood within three days of the first application [21]. It has also been approved as a second-line treatment for neuromyelitis optica and NMDAR encephalitis by the National Health Service (NHS) England [17]. A study indicated an increase in RTX use for pediatric CNS inflammatory disorders, particularly AEs [22]. In an anti-NMDAR encephalitis study, patients who received RTX immunotherapy had better outcomes at two years than those who did not receive second-line therapy [23].

Case reports by Paula Carrascosa-García highlighted the favourable response of two infants with anti-NMDAR encephalitis postherpes simplex encephalitis to RTX, suggesting its feasibility in infants. However, more data are needed on the long-term effects of RTX on B cell depletion and immunoglobulin levels in young infants [24]. For pediatric patients with GAD encephalitis, RTX is more commonly used than in other antibody subtypes of AE [25]. A prospective study of 10 patients with anti-NMDAR encephalitis suggested that low-dose RTX (100 mg IV weekly for 4 weeks) resulted in good outcomes in 9 patients, including 3 with full recovery and 1 patient with relapse [26].

RTX is generally well tolerated, with potential side effects, including infusion reactions, early-onset neutropenia (within one month) and late-onset neutropenia (up to one year, usually around 4-6 months), which can be self-limited, hypogammaglobulinemia, and the risk of opportunistic infections [21]. Therefore, monitoring blood counts, lymphocyte panels, and immunoglobulins during RTX treatment is necessary.

### Cyclophosphamide (CP)

CP is an alkylating agent that exerts cytotoxic effects by cross-linking DNA strands and inhibiting protein synthesis [27]. It has been used as an antineoplastic agent for more than 40 years. In addition to its antimitotic effects, CP effectively inhibits humoral immunity mediated by B lymphocytes and cell-mediated immunity by depleting T cells, making it widely used in the treatment of various autoimmune diseases [28]. Compared with RTX, CP is more cost-effective and convenient [29]. In a case study by Kashya Pethree et al., three girls (aged 27 months to 14 years) with anti-NMDAR encephalitis showed no consistent or sustained clinical improvement after first-line therapy. However, they exhibited dramatic clinical improvement when treated with monthly cycles of CP [30]. One patient with anti-GAD encephalitis and refractory status epilepticus achieved remarkable and sustained seizure control after CP treatment [31]. However, CP has numerous adverse effects [32]. Short-term adverse effects include infection, alopecia, and dermatitis, whereas long-term adverse effects include malignancy, infertility, and gonadal failure.

### Mycophenolate mofetil (MMF) and azathioprine (AZA)

For patients with a severe course or relapsing disease, maintenance immunosuppression with MMF and AZA may be beneficial. Generally, the duration of maintenance immunotherapy should be at least 12 months. MMF and AZA are immunosuppressants that are extensively used to prevent organ transplant rejection and to treat various autoimmune disorders. The efficacy and safety of MMF in treating AE has been documented in numerous studies [33–36]. Some clinicians initiate the use of steroid-sparing medications at the onset of AE to reduce reliance on steroids or IVIG without causing relapse [37]. Additionally, MMF and AZA are administered to patients who respond positively to RTX but experience rapid B-cell regrowth, providing additional protection [38].

There are currently no trials comparing the effects of second-line therapies. However, based on previous case reports, RTX has shown better efficacy and fewer side effects. CP has the advantage of lower cost, but has more side effects. CP is preferred to rituximab in paraneoplastic disorders, probably because of its bioavailability in the CNS, antimitotic effects and T cell depletion. Oral agents (MMF and AZA) are convenient to use and may be helpful in patients who respond well to first-line therapies but have difficulty tapering steroids or IVIG, or who are at risk of relapse. MMF is a safer oral immunosuppressant with a strong inhibitory effect on lymphocytes, a low incidence of adverse effects and is more convenient to administer than RTX, which requires hospitalisation for infusion.

## Third-line therapy

Although first-line and second-line therapies are effective for many patients with AE, a minority do not respond and are labelled “refractory”. These patients typically experience high disability, prolonged intensive care unit admissions, and high relapse rates. In these cases, more effective treatments are needed.

### Plasma cell‑depleting agents

#### Bortezomib (BTZ)

Firstly, we need to review the role of B cells and plasma cells in AE, as it is mainly caused by the production of pathogenic anti-neuronal antibodies mediated by humoral immunity [39]. During the process of further differentiation of precursor B lymphocytes and mature B lymphocytes into plasma cells, the expression of CD20 surface antigen gradually decreases and there is no expression of the CD20 on plasma cells. RTX is a monoclonal antibody that only targets only CD20 + B cells. Before the use of RTX, a large number of plasma cells have already been produced in the patient’s body. Short-lived plasma cells (SLPCs) derived from activated B cells can be depleted by RTX, but long-lived plasma cells (LLPCs) differentiated from B cells in the germinal centre can migrate to the bone marrow and continue to produce pathogenic antibodies, affecting the autoimmune state and leading to unrelieved clinical symptoms, and can be resistant to conventional immunosuppressive, B cell depletion therapy and anti-proliferative drugs such as cyclophosphamide. In addition, RTX cannot cross the blood-brain barrier and cannot eliminate intraspinal B cells [40, 41].

BTZ is a highly selective and reversible inhibitor of the 26S proteasome [42]. It targets cells with high protein synthesis, such as plasma cells, leading to apoptotic cell death by inhibiting proteasome function. While it is widely accepted that bortezomib typically does not penetrate the blood-brain barrier under normal conditions, it is plausible that in chronic brain inflammation and impaired blood-brain barrier, proteasome inhibition may reach both intrathecal and parenchymal plasma cells within the central nervous system [43]. BTZ may improve clinical outcomes for some patients with refractory AEs who do not respond to RTX, with an onset time of 2 to 3 weeks, making it a viable alternative for refractory cases. The combination of BTZ and RTX not only depletes both short-lived and long-lived plasma cells, but also prevents the production of new autoreactive B cells and plasma cells and the migration of plasma cell precursors into the brain [44–46]. Studies have shown that BTZ can deplete antibody-secreting cells that are insensitive to RTX, thereby achieving therapeutic effects [47].

A systematic review including 29 patients diagnosed with refractory anti-NMDAR encephalitis revealed that 16 patients (55.2%) experienced favourable outcomes after one to six cycles of BTZ [48]. A case-control study from China revealed that approximately half of the patients with anti-NMDAR encephalitis resistant to RTX experienced clinical improvement after receiving BTZ. The antibody titre and the number of plasma cells in the blood significantly decreased, with no serious adverse reactions, and no relapses were observed during a follow-up period of 31 months [47]. Johannes Wischmann et al. reported a patient with anti-Septin-5 encephalitis who initially responded temporarily to corticosteroids, PE, and RTX but relapsed. Upon reinitiation of PE and subsequent administration of BTZ, the patient achieved a moderate but sustained clinical improvement [49]. In isolated case reports or small-scale case studies, BTZ was used as a subsequent option after the ineffectiveness of standard and nonstandard therapies [45, 46, 50–55]. However, a prospective study did not establish the impact of BTZ on disease progression compared with an untreated historical control group. This result might be partially due to insufficient penetration of the blood-brain barrier by the BTZ, and the majority of these patients have high levels of anti-NMDAR antibodies in the CSF, which is often linked to a less favourable prognosis [56]. Potential side effects of BTZ include infections, digestive system disturbances, peripheral nerve damage, and a decrease in blood cell counts [42].

#### Daratumumab (DARA)

DARA is a human monoclonal antibody of the IgG1 subclass that targets plasma cells and plasma blasts expressing CD38. Scheibe et al. reported a case of a 60-year-old patient with refractory, aggressive anti-CASPR2 encephalitis characterized by high T cell activation and elevated anti-CASPR2 antibody levels. Standard treatments, including MP, PE, immunoadsorption, IVIG, and RTX, were ineffective. The treatment was escalated to 13 rounds of DARA at 16 mg/kg, resulting in significant neurological improvement. This positive outcome was attributed to DARA’s ability to deplete autoreactive, long-lived plasma cells, reducing anti-CASPR2 antibody levels in the CSF [57]. A case report by Dominica Ratuszny described the successful use of DARA in treating anti-NMDAR encephalitis unresponsive to conventional therapies [58]. A retrospective case series of five patients also supported the role of DARA in treating refractory AE, suggesting that DARA depletes long-lived plasma cells more effectively than BTZ does [59]. Adverse effects of DARA were documented in a systematic review of 83 patients, 45% of whom experienced adverse events, including application-related reactions (20%), infections (19%), and hypogammaglobulinemia (33%) [60]. A practical suggestion for clinical practice is to initiate DARA treatment with a prudent interval from previous immunotherapies to reduce the risk of critical infections due to overuse of immunosuppressants in a short period of time.

#### Obinutuzumab

Obinutuzumab, a type II anti-CD20 monoclonal antibody, is believed to cause more extensive depletion of B cells than type I anti-CD20 monoclonal antibodies such as RTX. In a case report, obinutuzumab showed promise as a safe and effective treatment option for individuals with ANCA-associated vasculitis, especially those who experienced treatment resistance or allergic reactions to RTX [61]. Obinutuzumab has been used to treat myasthenia gravis, but its application in treating AE is relatively limited. A study indicated that obinutuzumab offers superior biological effectiveness compared with RTX, with a longer duration before B cell repopulation. In this study, eight children (seven diagnosed with AE and one with myeloradiculitis) received obinutuzumab following RTX treatment to increase the effectiveness of anti-CD20 therapies and avoid high doses of RTX. After a single course of obinutuzumab, the median time for B cell repopulation was 230 days, ranging from 66 to 303 days, which was significantly longer than the 87 days observed after RTX. No adverse side effects were reported during obinutuzumab treatment, and all patients had positive outcomes at the most recent follow-up [62]. Common side effects of obinutuzumab include infusion reactions, neutropenia, thrombocytopenia, anaemia, fever, cough, and musculoskeletal disorders. Further research is needed to confirm the efficacy and safety of obinutuzumab in treating AEs.

### Cytokine‑based drugs

#### Tocilizumab (TCZ)

TCZ is a humanized monoclonal antibody that targets the IL-6 receptor, effectively inhibiting IL-6 signal transduction pathways. Tocilizumab can bind to both the soluble and membrane-bound IL-6 receptor, and exert several immunomodulatory effects. These effects include suppressing the activation of B cells, hindering the differentiation of cytotoxic T cells, curbing the proliferation of IL-17-producing Th17 cells, and fostering the differentiation of regulatory T cells. Notably, plasma cells rely on IL-6 for their survival, and tocilizumab is capable of disrupting the IL-6-dependent survival mechanisms in these cells [63]. In 2021, international guidelines for anti-NMDAR encephalitis recommended the addition of second-line immunotherapy for children with AE who do not respond to first-line immunotherapy after two weeks. If second-line immunotherapy is also ineffective within 1 to 3 months, TCZ treatment should be considered [64].

A prospective study of 78 patients with anti-NMDAR encephalitis revealed that 30 out of 52 patients who received TCZ treatment had a good prognosis. During follow-up, the most common adverse reaction was pulmonary infection, with a minority of patients experiencing neutrophil reduction, but most patients tolerated the treatment well [63]. In a study of 91 patients with AE who did not achieve adequate remission after RTX treatment, a retrospective analysis revealed that among the 30 patients who received TCZ, 89.5% experienced improvement in clinical symptoms within one month and maintained a favourable long-term clinical response [65].

In a case reported by Jang et al., a 6-year-old girl diagnosed with anti-LGI1 encephalitis who did not respond to first-line immunotherapy and RTX treatment experienced significant improvements in FBDSs and cognitive symptoms after being treated with TCZ [66]. Additionally, two patients with CASPR2 encephalitis and one patient with GAD65-associated encephalitis showed immediate and sustained clinical improvements, along with a decrease in antibody levels, after receiving TCZ as initial therapy [67–69]. TCZ could serve as a viable therapeutic alternative for individuals with myelin oligodendrocyte glycoprotein (MOG) antibody-associated encephalitis [70].

#### Anakinra

Anakinra, a synthetic form of the interleukin-1 receptor antagonist (IL-1Ra), has been approved by the European Medicines Agency (EMA) for treating various inflammatory conditions, including rheumatoid arthritis (RA), cryopyrin-associated periodic syndrome (CAPS), and Still’s disease. It works by blocking the action of IL-1, a proinflammatory cytokine, thus reducing inflammation and alleviating symptoms associated with these disorders [71]. In a rodent model simulating the passive transfer of anti-NMDAR encephalitis, characterized by seizures and cognitive impairments, anakinra treatment led to a rapid decrease in seizure activity within 24 to 48 hours, improved memory function, and a notable reduction in indicators of microglial and astrocytic activation [72]. Additionally, anakinra has been reported to significantly reduce seizure frequency in Rasmussen’s encephalitis patients [73, 74]. It has also been effectively used in treating new-onset refractory status epilepticus and febrile infection-related epilepsy syndrome [75, 76]. Thus, anakinra may serve as a potential therapeutic alternative for patients with epilepsy who are unresponsive to standard medications.

#### Tofacitinib

The JAK/STAT signalling pathway is crucial for proper immune system function and influences key components, such as cytokine receptors, inflammatory cytokines, and the regulation of T-helper cells. Tofacitinib, a drug that inhibits the JAK enzymes JAK1, JAK2, and JAK3, has been recognized as a pioneering treatment for RA. It was first approved in the United States and later in Japan, marking a significant advancement in the field of targeted synthetic disease-modifying anti-rheumatic drugs (tsDMARDs) [77, 78].

Tofacitinib has the ability to cross the blood-brain barrier, suggesting its potential efficacy in addressing autoimmune processes within the central nervous system. In a study on refractory AE, eight patients were treated with tofacitinib. Two individuals experienced significant improvement in their neurological conditions and brain imaging results. This included the resolution of chronic autoimmune meningoencephalitis and the termination of new-onset refractory status epilepticus in MOG antibody-related disorders, which had previously been resistant to sedatives and various other immunotherapeutic approaches [79].

#### Interleukin-2 (IL-2)

IL-2 can influence the growth and specialization of various immune cell subsets in a dose-dependent manner. High doses of IL-2 promote the maturation and proliferation of effector and memory T cells, whereas lower doses support the development, longevity, and functionality of regulatory T (Treg) cells, a subset of CD4 + T cells crucial for maintaining immune balance and tolerance. Consequently, IL-2 can have both stimulatory and suppressive effects on the immune system in the context of autoimmune disorders [80]. Clinical improvements with low-dose IL-2 have been observed in the treatment of systemic lupus erythematosus, rheumatoid arthritis, Behcet’s disease, ulcerative colitis, and other conditions [81]. In a retrospective analysis of 10 patients with refractory AEs who received low-dose IL-2 therapy, six patients showed improvement at the last follow-up compared with the start of treatment. This research suggests that low-dose IL-2 is a viable therapeutic approach for refractory AE [82].

### Treatments targeting intrathecal immune cells

Recent research suggests that the inadequate permeability of the blood-brain barrier to immunosuppressive drugs contributes to the ineffectiveness of primary and secondary treatments for AE. These findings underscore the need for the administration of immunosuppressive drugs, such as intrathecal methotrexate, directly into the CSF. Methotrexate, an anti-folate compound approved by the FDA, is used for both chemotherapy and immunosuppression in autoimmune diseases. Its intrathecal administration results in elevated CSF levels while minimizing systemic side effects [83]. Intrathecal methotrexate has been shown to be effective in case series and preliminary studies, mainly involving patients with refractory anti-NMDAR encephalitis [37, 84–86]. However, future extensive cohort studies and animal-based researches are needed to gain a deeper understanding of the benefits and underlying mechanisms of intrathecal methotrexate administration.

## Discussion

The existing evidence on treatment options for AE has many limitations, as it relies heavily on case reports or series, and a small number of controlled studies with relevant biases. No robust clinical trials have been conducted to evaluate and compare the different approaches within acute immunotherapy for AE. Currently, most trials and case reports lack a control group, making it impossible to distinguish between spontaneous improvement and treatment response. In addition, the mRS is the most commonly reported outcome measure in the literature for patients with AE. In a systematic review and individual patient data meta-analysis, no association was found between treatment with second-line immunotherapy and lower final mRS scores in patients with AE. This may reflect the insensitivity of the mRS to cognitive impairment at follow-up or its poor specificity in identifying severe cases of AE [87]. Therefore, a more appropriate scale such as the Clinical Assessment Scale in Autoimmune Encephalitis (CASE) should be adopted to overcome the limitations of current outcome scales for AE [88]. At the same time, antibody specificity, prior treatment, length of follow-up and treatment protocols also hinder the accurate assessment of treatment efficacy and the ability to compare various third-line therapeutic options.

The Autoimmune Encephalitis Alliance Clinicians Network (AEACN) survey suggested the best therapeutic recommendations for AE. Corticosteroids, either alone or in combination with other treatments such as IVIG or PE, were chosen as the first-line treatment by 84% of respondents for patients with a typical presentation. Regarding the preferred second-line treatment, 80% of respondents opted for RTX, whereas only 10% selected CP in a clinical context where antibody status was unknown. In the absence of clear objective or subjective signs of improvement with standard second-line treatments, one might consider exploring innovative therapies like tocilizumab or bortezomib, despite the limited evidence of their efficacy [5]. A similar recommendation has been proposed to standardise the treatment of paediatric NMDARE, and tocilizumab appears to be more popular due to a more favourable perceived safety profile [64].

There are currently several promising randomised clinical trials in AE. A multicentre, randomised, controlled, double-blind, phase II trial is underway to evaluate the efficacy and safety of bortezomib in patients with severe autoimmune encephalitis who have failed to respond to rituximab(NCT03993262) [43]. And the phase III, randomised, double-blind, multicentre, basket trial (NCT05503264) will evaluate the efficacy of satralizumab in NMDAR or LGI1AE [89].

## Conclusion

This article systematically reviews the management of AE, addressing first-, second-, and third-line therapies, with special emphasis on innovative drugs for resistant AE. First-line therapies include IVMP, IVIG, and PE. If there is no clear clinical improvement within 2-3 weeks, second-line options such as RTX or CP should be initiated immediately. Third-line therapies, such as TCZ and BTZ, are becoming more popular and are the focus of ongoing studies, although more evidence is needed. Randomised, prospective, placebo-controlled trials are needed to further evaluate the efficacy of different treatment approaches.
